# Prognostic factors in patients with septic disseminated intravascular coagulation treated with thrombomodulin: the effect of reduced thrombomodulin dose; a single-center, retrospective, observational study

**DOI:** 10.1186/s40780-022-00264-9

**Published:** 2022-12-12

**Authors:** Yoshihiro Nishita, Masatoshi Taga, Masaru Sakurai, Yoshitsugu Iinuma, Togen Masauji

**Affiliations:** 1 Department of Pharmacy, Kanazawa Medical University Hospital, 1-1 Daigaku, Uchinada-cho, Kahoku-gun, Ishikawa 920-0293 Japan; 2 Department of Social and Environmental Medicine, Kanazawa Medical University, 1-1 Daigaku, Uchinada-cho, Kahoku-gun, Ishikawa 920-0293 Japan; 3 Department of Infectious Disease, Kanazawa Medical University, 1-1 Daigaku, Uchinada-cho, Kahoku-gun, Ishikawa 920-0293 Japan

**Keywords:** Human soluble thrombomodulin alfa, Sepsis, Disseminated Intravascular Coagulation (DIC), Dose reduction, DIC resolution

## Abstract

**Background:**

Human soluble recombinant thrombomodulin (TM alfa), a treatment for septic Disseminated intravascular coagulation (DIC), is recommended for patients with severe renal dysfunction in reduced doses. However, no studies have examined yet how dose reduction affects clinical efficacy. In this study, we investigated the significance of the TM alfa dose as a prognostic factor in clarifying the clinical background factors related to the clinical effect of TM alfa in patients with septic DIC.

**Methods:**

This study involved 102 patients with septic DIC admitted to a single-center intensive care unit between April 2013 and March 2020, receiving TM alfa. The following factors were retrospectively collected from the medical records of the target patients: (1) patient background, (2) sequential organ failure assessment (SOFA) score, (3) Japanese Association for Acute Medicine DIC diagnostic criteria score, (4) DIC treatment information, (5) TM alfa dose per bodyweight (normal dose: 0.06 mg/kg or reduced dose: 0.02 mg/kg), (6) DIC resolution within 7 days after the start of TM alfa administration (DIC resolution), (7) all deaths within 30 days after the start of TM alfa administration (30-days-all-cause mortality), (8) presence or absence of new hemorrhagic side effects after the start of TM alfa administration. Multiple logistic regression analysis was used to assess factors associated with DIC resolution and 30-days-all-cause mortality.

**Results:**

The SOFA score (odds ratio: 95% confidence interval, 0.76: 0.66–0.89), pneumonia (0.24: 0.08–0.75), and reduced dose administration of TM alfa (0.23: 0.08–0.66) were independent of and negatively related to the DIC resolution. For the 30-days-all-cause mortality, the SOFA score (1.66: 1.31–2.09), pneumonia (9.50: 2.49–36.25), and TM alfa dose reduction (3.52: 1.06–11.69) were independent, poor prognostic factors. We found no association between the hemorrhagic side effects and the TM alfa dose per bodyweight.

**Conclusions:**

The reduced dose of TM alfa for patients with severe renal dysfunction was observed to be an influential factor for DIC resolution and 30-day all-cause mortality, as were SOFA scores and pneumonia. Further studies are required in the future to verify this finding.

## Background

Sepsis is a major cause of intensive care unit (ICU) admission in the developed countries, including Japan, and is a condition with a high mortality rate [1, 2]. The mortality rate is reportedly higher in patients with disseminated intravascular coagulation (DIC) [3–5], although the treatment of patients with DIC has yet not been properly established.

Thrombomodulin (TM) alfa, a recombinant human soluble thrombomodulin, is an anticoagulant drug approved and introduced in Japan in 2008 for the treatment of DIC [6]. Like thrombomodulin, TM alfa not only directly inhibits the coagulation activity of thrombin by forming a complex with it but also helps to activate protein C, an anticoagulant factor, and to inactivate activated factor V and factor VIII, thereby inhibiting blood coagulation [6, 7]. In addition, TM alfa is frequently used for sepsis-associated DIC due to its lectin-like domain-mediated anti-inflammatory effects, recent meta-analyses proving its clinical efficacy [8, 9]. Therefore, the Surviving Sepsis Campaign Guidelines [10] and the Japanese Sepsis Guidelines 2016 did not provide a clear recommendation for TM alfa in patients with septic DIC, although the latest Japanese Sepsis Guideline 2020 weakly recommends TM alfa in patients with septic DIC.

The urinary excretion rate of TM alfa within 48 hours after administration has been reported to be 54.3–59.8% [11]. TM alfa is usually used at a dose of 0.06 mg/kg (380 U/kg) but in patients with severe renal dysfunction, the dose could be reduced to 0.02 mg/kg (130 U/kg) according to the symptoms. Nevertheless, Hayakawa et al. reported no difference in the TM alfa blood concentration depending on the dosage between patients with Creatinine clearance (CCr) less than 10 mL/min and those with CCr greater than 10 mL/min [12]. Therefore, the effective blood concentration might not be reached at a dose of 0.02 mg/kg of TM alfa in patients with renal dysfunction. Imaura et al. reported a significantly higher 90-day survival rate in patients with plasma levels of TM alfa higher than 600 ng/mL [13]. Therefore, reaching an effective TM alfa plasma concentration seems to be important for improving clinical outcomes. However, no currently available reports have examined how the different doses (0.06 or 0.02 mg/kg) could affect clinical efficacy in patients with severe renal dysfunction.

In this study, we aimed at clarifying the clinical background factors associated with the prognosis of patients with septic DIC treated with TM alfa, such as DIC resolution, death, and the occurrence of hemorrhagic complications. Based on the results of these analyses, we discussed the significance of the TM alfa dose as a prognostic factor.

## Methods

This was a single-center, retrospective study of adult patients with septic DIC treated with TM alfa in the ICU of Kanazawa Medical University Hospital, approved by the Ethical Review Committee of Kanazawa Medical University Hospital (Approval number: H263). Due to the retrospective nature of the study, informed consent was waived by this committee. The study was conducted in accordance with Good Clinical Practice (Declaration of Helsinki).

### Patients

This study was conducted between April 1, 2013 and March 31, 2020, including patients admitted to the mixed medical ICU and surgical ICU of Kanazawa Medical University Hospital, with a total of 18 beds. We included in the study those patients who received TM alfa and fulfilled the following criteria: adult patients aged at least 20 years, selected using the Sepsis-3 diagnostic criteria (suspected infection and a sudden increase in Sequential Organ Failure Assessment (SOFA) score of more than 2 points), and the Japanese Association for Acute Medicine DIC diagnostic criteria (JAAM-DIC) score of at least 4 points. We established two dosage groups for TM alfa: (1) 0.06 mg/kg with or without severe renal dysfunction [CCr < 15 mL/min or oliguria (urine output < 0.5 mL/kg/hr)] (normal dose group) and (2) 0.02 mg/kg for severe renal dysfunction (reduced dose group). However, doses set at ±5 kg bodyweight were included in the analysis of this study. The following patients were excluded: (1) patients who did not meet the criteria for JAAM-DIC diagnosis immediately before TM alfa administration, (2) patients younger than 20 years, (3) patients receiving a dose set different from 0.02 mg/kg or 0.06 mg/kg, (4) patients with no decline in renal function (CCr ≥ 15 mL/min and urine output ≥0.5 mL/kg/hr) and with a dose set 0.02 mg/kg, (5) patients with a TM alfa dose changed during the DIC treatment, (6) hematology patients with a platelet count consistently lower than 80,000/μL, (7) patients with a treatment period shorter than 2 days.

### Variables and outcomes

The following immediately-before-TM alfa-administration factors were investigated retrospectively from the medical records: patient background; age, sex, height, weight, CCr, presence of severe renal dysfunction, blood urea nitrogen, presence of renal replacement therapy, presence of malignancy, serum albumin, serum antithrombin III (AT III), C reactive protein, type of infection, SOFA score, JAAM-DIC score, systemic inflammatory response syndrome (SIRS) criteria. The following factors were investigated as parts of the DIC treatment: antimicrobial agents, AT III agent, fresh frozen plasma (FFP), platelet concentrate (PC), sodium heparin (except for low-dose sodium heparin for deep vein thrombosis prophylaxis and saline with heparin for line keeping), TM alfa dose per bodyweight (0.06 or 0.02 mg/kg), and Polymyxin B immobilized fiber column direct hemoperfusion (PMX-DHP). FFP and PC were used according to the “Guidelines for the Use of Blood Products” in Japan. Particularly, FFP was used after major surgery or when bleeding risk was high (PT-INR ≥ 2.0, APTT ≥2 times the upper limit of our standard, and fibrinogen level was lowered or likely to be lowered below 150 mg/dL). Conversely, PC was used to raise the platelet count above 50,000/μL in post-surgical or platelet counts below 50,000/μL. The following outcome factors were investigated: DIC resolution within 7 days of TM alfa initiation, 30-days-all-cause mortality from TM alfa initiation, and the presence and details of any new bleeding side effects 30 days after TM alfa initiation (bleeding site, whether the bleeding was major bleeding according to the International Society of Thrombosis and Hemostasis bleeding criteria) [14]. Since DIC resolution is a prevention measure of the development of multiorgan damage by improving vascular endothelial damage, and mortality is a measure of short-term prognosis, each outcome was examined. Based on past studies, the resolution of DIC was defined as a JAAM-DIC score of fewer than 4 points [15, 16]. DIC resolution group was defined as DIC resolution within 7 days of TM alfa initiation, and the non-DIC resolution group was defined as a JAAM-DIC score of 4 points or more after 7 days of TM alfa initiation.

### Statistical analysis

We exploratively examined the clinical factors associated with each outcome: DIC resolution within 7 days of starting TM alfa, 30-days-all-cause mortality from TM alfa initiation, and new bleeding complications after TM alfa initiation. The baseline attributes and treatment methods were compared between the patients with and without each outcome. Age, sex, and severity of illness were compared between the normal dose group and the reduced dose group.

The data are presented as the median (interquartile range) or number (%). The chi-square test or Fisher’s exact test were performed for univariate analysis of categorical variables, and the Mann–Whitney *U* test was performed for univariate analysis of the continuous variables. Factors associated with DIC resolution within 7 days of TM alfa initiation and 30-days-all-cause mortality were evaluated using multiple logistic regression analysis. The objective variable was the occurrence of each outcome. For the explanatory variables, all baseline subject attributes associated with the outcome (*p* < 0.1) were entered into the model, and the relevant variables were selected using the stepwise (increasing variable) method. Confidence interval (CI) was calculated. For the outcome of hemorrhagic complications, the relevant background factors were evaluated by univariate analysis but the multivariate analysis was not performed for hemorrhagic complications due to the small number of cases.

We used the SPSS software version 27 (IBM Corp, Armonk, New York, USA) for statistical analysis, and two-sided *P*-values of *p* < 0.05 were considered statistically significant.

## Results

**Fig. 1:**
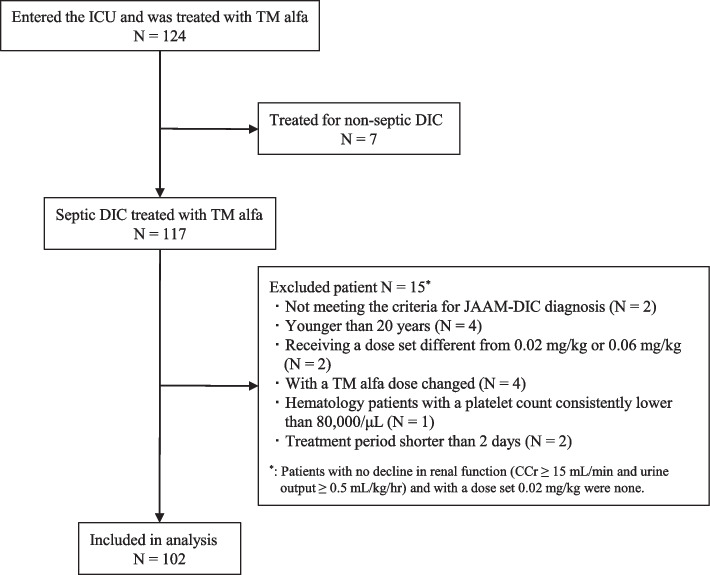
Patient flow diagram. *ICU* intensive care unit, *TM* thrombomodulin, *DIC* disseminated intravascular coagulation, *JAAM-DIC* Japanese Association for Acute Medicine DIC diagnostic criteria

A total of 102 patients who received TM alfa during the study period were included in the study (Fig. 1). Table 1 shows the patient background and Table 2 shows the characteristics of the TM dose. There were no significant differences between TM doses for all items examined.

**Table 1:** Patients’ baseline characteristics

	Total (***N*** = 102)
Patient characteristics	
Age, years	76 (67–83)
Male sex	58 (56.9)
Height, cm	1.58 (1.50–1.65)
Weight, kg	54.0 (44.8–63.8)
Body mass index	21.7 (19.1–25.1)
Creatinine clearance, mL/min	21.31 (12.35–39.85)
Severe renal dysfunction	54 (52.9)
Blood urea nitrogen, mg/dL	39.0 (27.3–57.8)
Renal replacement therapy	55 (53.9)
Malignancy	22 (21.6)
Serum albumin, mg/dL	2.35 (1.92–2.70)
C reactive protein, mg/dL	16.99 (9.32–24.10)
Illness severity	
SOFA score	11 (9–14)
JAAM-DIC score	6 (5–7)
SIRS criteria	3 (2–4)
Primary type of Infection	
Intra-abdominal infection	25 (24.5)
Pneumonia	23 (22.5)
Urinary tract infection	17 (16.7)
Cholangitis/Cholecystitis	9 (8.8)
Blood stream infection	7 (6.9)
Infectious enteritis	2 (2.0)
Necrotizing fasciitis/Gas gangrene	2 (2.0)
Osteomyelitis	2 (2.0)
Iliopsoas muscle abscess/Retroperitoneal abscess	2 (2.0)
Others^*a*^	4 (3.9)
Unknown	9 (8.8)
Treatment	
Dose reduction of TM alfa	30 (29.4)
Duration of TM alfa administration	6 (4–6)
Antibiotics	102 (100.0)
Antithrombin III	78 (76.5)
Heparin	3 (2.9)
Fresh frozen plasma	29 (28.4)
Platelet Concentrate	37 (36.3)
PMX-DHP	48 (47.1)
Clinical outcomes	
DIC resolution up to day 7	55 (53.9)
All-cause mortality at 30 days	30 (29.4)
Bleeding complications	13 (12.7)

Data are presented as median (interquartile range) or number (%)

*SOFA* Sequential organ failure assessment, *JAAM-DIC* Japanese Association for Acute Medicine DIC diagnostic criteria, *DIC* disseminated intravascular coagulation, *SIRS* Systemic inflammatory response syndrome, *PMX-DHP* Polymyxin B immobilized fiber column direct hemoperfusion

^*a*^Pulmonary abscess, Subcutaneous abscess, Incision infection, severe pancreatitis

**Table 2:** Characteristics Stratified by TM dose

	Normal dose group (***N*** = 72)	Reduced dose group (***N*** = 30)	***p*** value
Age, years	76 (29–92)	78.5 (21–95)	0.366^*a*^
Male sex	38 (52.8)	20 (66.7)	0.273^*b*^
SOFA score	11 (5–22)	12 (5–18)	0.285^*a*^
JAAM-DIC score	6 (4–8)	5 (4–8)	0.471^*a*^
SIRS criteria	3 (1–4)	3 (1–4)	0.672^*a*^

Data are presented as median (interquartile range) or number (%)

*SOFA* Sequential organ failure assessment, *JAAM-DIC* Japanese Association for Acute Medicine DIC diagnostic criteria, *DIC* disseminated intravascular coagulation, *SIRS* Systemic inflammatory response syndrome

^*a*^Mann-Whitney *U* test

^*b*^Chi-square test

Table 3 shows the relationship between the DIC resolution and clinical background. Compared with the DIC resolution group, the non-DIC resolution group contained significantly more males, just as well as patients with renal replacement therapy and significantly lowers CCr. The SOFA score was significantly higher in the non-DIC resolution group. Pneumonia was more common in the non-DIC resolution group, while cholangitis/cholecystitis was significantly more common in the DIC resolution group. A reduced TM alfa dose was more common in the non-DIC resolution group, and FFP was significantly less common in the non-DIC resolution group. The JAAM-DIC score, unknown type of infection, and PC administration tended to be associated with DIC resolution (*p* < 0.1). All the factors associated with the presence or absence of DIC resolution were entered into the model, and the factors associated with the outcome were selected by the logistic regression analysis stepwise method. As a result, three factors were selected: SOFA score, pneumonia, and reduced TM alfa dosage. These were found to be significantly associated with DIC resolution independently of each other (Table 4). That is, the higher the SOFA score, the lower the odds ratio (OR) for the DIC resolution (OR for DIC resolution per 1 increase in SOFA score, 0.76; 95% CI, 0.66–0.89). Pneumonia had a significantly lower OR for the DIC resolution (OR, 0.24; 95% CI, 0.08–0.75), and the TM alfa OR for the DIC resolution was significantly lower in the TM alfa reduced dose group compared with the normal dose group (OR, 0.23; 95% CI, 0.08–0.66).

**Table 3:** Baseline Patient’s Characteristics Stratified by DIC resolution

	DIC resolution		
	Yes (***N*** = 55)	No (***N*** = 47)	***p*** value
Patient characteristics			
Age, years	76 (66–84)	78 (69–83)	0.392^*a*^
Male sex	23 (41.8)	35 (74.5)	0.002^*b*^
Body mass index	21.7 (19.2–25.7)	21.7 (18.8–24.4)	0.582^*a*^
Creatinine clearance, mL/min	26.5 (13.1–49.1)	15.6 (11.8–31.0)	0.046^*a*^
Renal replacement therapy	24 (43.6)	31 (66.0)	0.040^*b*^
Malignancy	10 (18.2)	13 (25.5)	0.510^*b*^
Serum albumin, mg/dL	2.4 (2.0–2.8)	2.3 (2.0–2.7)	0.294^*a*^
Illness severity			
SOFA score	9 (8–12)	13 (10–16)	< 0.001^*a*^
JAAM-DIC score	5 (5–7)	6 (5–8)	0.053^*a*^
SIRS criteria	3 (2–4)	3 (3–4)	0.199^*a*^
Primary type of Infection			
Intra-abdominal infection	16 (29.1)	9 (19.1)	0.260^*c*^
Pneumonia	6 (10.9)	17 (36.2)	0.004^*c*^
Urinary tract infection	11 (20.0)	6 (12.8)	0.427^*c*^
Cholangitis/Cholecystitis	9 (16.4)	0 (0.0)	0.003^*c*^
Blood stream infection	3 (5.5)	4 (8.5)	0.701^*c*^
Infectious enteritis	2 (3.6)	0 (0.0)	0.498^*c*^
Necrotizing fasciitis/Gas gangrene	1 (1.8)	1 (2.1)	1.000^*c*^
Osteomyelitis	2 (3.6)	0 (0.0)	0.498^*c*^
Iliopsoas muscle abscess/Retroperitoneal abscess	1 (1.8)	1 (2.1)	1.000^*c*^
Unknown	2 (3.6)	7 (14.9)	0.077^*c*^
Treatment			
Dose reduction of TM alfa	9 (16.4)	21 (44.7)	0.002^*c*^
Antithrombin III	40 (72.7)	38 (80.9)	0.360^*c*^
Heparin	1 (1.8)	2 (4.3)	0.594^*c*^
Fresh frozen plasma	18 (38.3)	11 (20.0)	0.049^*b*^
Platelet Concentrate	15 (27.3)	22 (46.8)	0.066^*b*^
PMX-DHP	28 (50.9)	20 (42.6)	0.520^*b*^

Data are presented as median (interquartile range) or number (%)

*SOFA* Sequential organ failure assessment, *JAAM-DIC* Japanese Association for Acute Medicine DIC diagnostic criteria, *DIC* disseminated intravascular coagulation, *SIRS* Systemic inflammatory response syndrome, *PMX-DHP* Polymyxin B immobilized fiber column direct hemoperfusion

^*a*^Mann-Whitney *U* test

^*b*^Chi-square test

^*c*^Fisher’s exact test

**Table 4:** Multivariable logistic regression analyses for DIC resolution

	OR (95% CI)	*p* value
SOFA score	0.76 (0.66–0.89)	< 0.001
Pneumonia	0.24 (0.08–0.75)	0.014
Dose reduction of TM alfa	0.23 (0.08–0.66)	0.006

*OR* odds ratio, *CI* confidence interval, *SOFA* Sequential organ failure assessment

Table 5 shows the relationship between the presence of the 30-days-all-cause mortality and the clinical background. The SOFA score was significantly higher, pneumonia and TM alfa dose reduction were significantly more common, and PMX-DHP was significantly less common in the death group. Serum albumin and cholangitis/cholecystitis tended to be associated with the 30-days-all-cause mortality (*p* < 0.1). The 30-days-all-cause mortality was defined as an outcome, and the related factors were evaluated by logistic regression analysis. The results showed that a high SOFA score (OR, 1.66; 95% CI, 1.31–2.09), pneumonia (OR, 9.50; 95% CI, 2.49–36.25), and reduced TM alfa dose (OR, 3.52; 95% CI, 1.06–11.69) were independently associated with a significant increase in the OR (Table 6).

**Table 5:** Baseline Patient’s Characteristics Stratified by All-cause mortality at 30 days

	All-cause mortality at 30 days		
	Yes (***N*** = 30)	No (***N*** = 72)	***p*** value
Patient characteristics			
Age, years	74 (69–80)	77 (67–84)	0.683^*a*^
Male sex	21 (70.0)	37 (51.4)	0.124^*c*^
Body mass index	21.4 (17.0–24.2)	21.7 (19.2–25.5)	0.352^*a*^
Creatinine clearance, mL/min	21.3 (12.7–29.4)	20.6 (12.4–42.6)	0.523^*a*^
Renal replacement therapy	20 (66.7)	35 (48.6)	0.147^*b*^
Malignancy	8 (26.7)	14 (19.4)	0.437^*c*^
Serum albumin, mg/dL	2.15 (1.90–2.50)	2.45 (2.08–2.73)	0.093^*a*^
Illness severity			
SOFA score	15 (12–17)	10 (8–12)	< 0.001^*a*^
JAAM-DIC score	6 (5–7)	6 (5–7)	0.448^*a*^
SIRS criteria	3 (2–4)	3 (2–4)	0.832^*a*^
Primary type of Infection			
Intra-abdominal infection	4 (13.3)	21 (29.2)	0.129^*c*^
Pneumonia	14 (46.7)	9 (12.5)	< 0.001^*c*^
Urinary tract infection	2 (6.7)	15 (20.8)	0.142^*c*^
Cholangitis/Cholecystitis	0 (0.0)	9 (12.5)	0.055^*c*^
Blood stream infection	3 (10.0)	4 (5.6)	0.417^*c*^
Infectious enteritis	0 (0.0)	2 (2.8)	1.000^*c*^
Necrotizing fasciitis/Gas gangrene	0 (0.0)	2 (2.8)	1.000^*c*^
Osteomyelitis	1 (3.3)	1 (1.4)	0.504^*c*^
Iliopsoas muscle abscess/Retroperitoneal abscess	0 (0.0)	2 (2.8)	1.000^*c*^
Unknown	5 (16.7)	4 (5.6)	0.119^*c*^
Treatment			
Dose reduction of TM	14 (46.7)	16 (22.2)	0.026^*b*^
Antithrombin III	26 (86.7)	52 (72.2)	0.133^*c*^
Heparin	1 (3.3)	2 (2.8)	1.000^*c*^
Fresh frozen plasma	11 (36.7)	18 (25.0)	0.342^*b*^
Platelet Concentrate	12 (40.0)	25 (34.7)	0.780^*b*^
PMX-DHP	8 (26.7)	40 (55.6)	0.009^*c*^

Data are presented as median (interquartile range) or number (%)

*SOFA* Sequential organ failure assessment, *JAAM-DIC* Japanese Association for Acute Medicine DIC diagnostic criteria, *DIC* disseminated intravascular coagulation, *SIRS* Systemic inflammatory response syndrome, *PMX-DHP* Polymyxin B immobilized fiber column direct hemoperfusion

^*a*^Mann-Whitney *U* test

^*b*^Chi-square test

^*c*^Fisher’s exact test

**Table 6:** Multivariable logistic regression analyses for All-cause mortality at 30 days

	OR (95% CI)	*p* value
SOFA score	1.66 (1.31–2.09)	< 0.001
Pneumonia	9.50 (2.49–36.25)	0.001
Dose reduction of TM alfa	3.52 (1.06–11.69)	0.040

*OR* odds ratio, *CI* confidence interval, *SOFA* Sequential organ failure assessment

Bleeding adverse reactions were observed in 13 patients (12.7%) by day 7 after the completion of dosing (Table 7). The major sites of bleeding were of subcutaneous, oral, and gastrointestinal localization in three cases each, followed by bleeding from the site of vascular penetration in two cases. One major bleeding case of gastrointestinal bleeding was observed in a patient with reduced dosage on TM alfa. As for the factors associated with hemorrhagic adverse reactions (Table 8), infectious enteritis was significantly more common but no differences in other factors have been observed.

**Table 7:** Details of Bleeding complications

Details	***N*** = 13
Subcutaneous bleeding	3 (23.1)
Mouth hemorrhage	3 (23.1)^*a*^
Gastrointestinal hemorrhage	3 (23.1)^*b*^
Bleeding at the site of vascular access	2 (15.4)
Urinary hemorrhage	1 (7.7)^*a*^
Nasal hemorrhage	1 (7.7)
Alveolar hemorrhage	1 (7.7)

^*a*^same

^*b*^one case serious bleeding

**Table 8:** Baseline Patient’s Characteristics Stratified by Bleeding complications

	Bleeding complications		
	Yes (***N*** = 13)	No (***N*** = 89)	***p*** value
Patient characteristics			
Age, years	71 (59–80)	77 (68–83)	0.164^*a*^
Male sex	8/5	50/39	0.773^*c*^
Body mass index	20.2 (18.1–21.8)	22.0 (19.2–25.5)	0.074^*a*^
Creatinine clearance, mL/min	14.1 (12.3–39.9)	22.3 (12.4–39.7)	0.479^*a*^
Renal replacement therapy	7 (53.8)	48 (53.9)	1.000^*c*^
Malignancy	3 (23.1)	19 (21.3)	1.000^*c*^
Serum albumin, mg/dL	2.40 (2.10–2.50)	2.30 (1.90–2.70)	0.687^*a*^
Illness severity			
SOFA score	13 (9–14)	11 (9–14)	0.453^*a*^
JAAM-DIC score	6 (5–7)	6 (5–7)	0.723^*a*^
SIRS criteria	3 (3–4)	3 (2–4)	0.566^*a*^
Primary type of Infection			
Intra-abdominal infection	3 (23.1)	22 (24.7)	1.000^*c*^
Pneumonia	4 (30.8)	19 (21.3)	0.483^*c*^
Urinary tract infection	1 (7.7)	16 (18.0)	0.690^*c*^
Cholangitis/Cholecystitis	0 (0.0)	9 (10.1)	0.599^*c*^
Blood stream infection	0 (0.0)	7 (7.9)	0.591^*c*^
Infectious enteritis	2 (15.4)	0 (0.0)	0.015^*c*^
Necrotizing fasciitis/Gas gangrene	0 (0.0)	2 (2.2)	1.000^*c*^
Osteomyelitis	0 (0.0)	2 (2.2)	1.000^*c*^
Iliopsoas muscle abscess/Retroperitoneal abscess	0 (0.0)	2 (2.2)	1.000^*c*^
Unknown	3 (23.1)	6 (6.7)	0.087^*c*^
Treatment			
Dose reduction of TM	3 (23.1)	27 (30.3)	0.751^*c*^
Antithrombin III	11 (84.6)	67 (75.3)	0.728^*c*^
Heparin	0 (0.0)	3 (3.4)	1.000^*c*^
Fresh frozen plasma	6 (46.2)	23 (25.8)	0.186^*c*^
Platelet Concentrate	6 (46.2)	31 (34.8)	0.539^*c*^
PMX-DHP	5 (38.5)	43 (48.3)	0.564^*c*^

Data are presented as median (interquartile range) or number (%)

*SOFA* Sequential organ failure assessment, *JAAM-DIC* Japanese Association for Acute Medicine DIC diagnostic criteria, *DIC* disseminated intravascular coagulation, *SIRS* Systemic inflammatory response syndrome, *PMX-DHP* Polymyxin B immobilized fiber column direct hemoperfusion

^*a*^Mann-Whitney *U* test

^*b*^Chi-square test

^*c*^Fisher’s exact test

## Discussion

Although septic DIC is a disease with a poor prognosis [3–5], few drugs have been proven clinically effective for its treatment. TM alfa is weakly recommended in the Japanese guideline for sepsis 2020, and several reports have been published on the efficacy of TM alfa in septic DIC [15, 17–19]. Furthermore, a recent meta-analysis has shown the benefit of TM alfa in septic DIC resolution and 28-days-all-cause mortality [8, 9]. Therefore, TM alfa has become one of the options for septic DIC. However, some of the reports in the meta-analysis excluded patients with severe renal dysfunction [16, 20, 21], and the efficacy of TM alfa at a reduced dose of 0.02 mg/kg has not been properly addressed either. Therefore, in this study, the factors affecting clinical efficacy in patients with septic DIC, including TM alfa dose were investigated.

In this study, despite the lack of significant differences in the severity of illness between TM doses, a reduced TM alfa dose was identified as a factor associated with the resolution of septic DIC and 30-days-all-cause mortality. These results indicate that TM alfa should be administered at a dose of 0.06 mg/kg regardless of renal function to maintain effective plasma concentrations and achieve full efficacy. In recent years, certain studies reported on the relationship between TM alfa pharmacokinetics and its clinical effects. For example, TM alfa plasma concentrations were reportedly similar in patients with different renal functions (CCr < 10 mL/min vs 10 mL/min ≤ CCr < 30 mL/min vs 30 mL/min ≤ CCr < 60 mL/min vs 60 mL/min ≤ CCr) [12]. In addition, TM alfa is a renal excretory drug, and the 24-hour urinary excretion rate correlates with CCr. Moreover, it has been pointed out that other metabolic processes, such as activated elastase-mediated degradation, might have a compensatory effect on the disappearance of TM alfa in patients with DIC and renal dysfunction [22, 23]. Another study on a case of septic DIC with a TM alfa plasma concentration below 600 ng/mL reported that the decrease in the JAAM-DIC score from the baseline 4 days after TM alfa administration and the 90-day survival rate were significantly higher and lower, respectively [13]. Therefore, the TM alfa dosage in patients with severe renal dysfunction might be important in improving the prognosis of patients with septic DIC.

In this study, the SOFA score was a proven independent factor associated with the resolution of DIC and 30-days-all-cause mortality. The SOFA score has been adopted as a diagnostic criterion for sepsis as an indicator of organ damage severity [24]. In a previous study, the SOFA score was reportedly associated with the 28-day mortality in patients with DIC [5]. Therefore, organ damage severity in patients with DIC could be related to prognosis. In addition, when patients with DIC were classified by the SOFA score, TM alfa reportedly improved the survival in a high-risk subset with a SOFA score of 13–17 [19]. In addition to the high risk of death in patients with a high SOFA score, a reduced TM alfa dose was identified as a factor associated the clinical outcomes. Therefore, the TM alfa dosage should be determined more carefully.

In this study, the most common sites of infection were the abdomen, lung, and urinary tract, respectively. Interestingly, in the Japanese Sepsis Registry study, the most common sites of infection causing sepsis were the lung, abdomen, and urinary tract, respectively [3]. In a study of Japanese ICUs, the most common sites of infection causing sepsis were the lung, abdomen, and urinary tract, respectively [1]. In an international study, respiratory, intra-abdominal, and bloodstream infections were the most common infectious sites in the ICU [25]. In the present study, intra-abdominal infection was the most common, although the infected sites were similar to those in the previous studies of sepsis. This might be due to the fact that intra-abdominal infections have a higher risk of developing into DIC [26] since they are often subjected to stress due to surgery in addition to organ damage caused by infection. In the present study, pneumonia was associated with DIC resolution and 30-day all-cause mortality, indicating that pneumonia-associated DIC might be an independent factor in mortality regardless of the TM alfa dose. In the case of sepsis, pneumonia reportedly exhibited a higher in-hospital mortality rate than in the case of intra-abdominal and urinary tract infections [3]. Moreover, a retrospective Japanese nationwide study [27] reported that TM alfa did not improve the 28-day mortality in pneumonia-associated DIC. The usefulness of TM alfa in the treatment of pneumonia-associated DIC needs to be further investigated.

In this study, the incidence of major bleeding was 1.0% (only one case of bleeding occurred in a patient with reduced dosage), and the overall incidence of bleeding was 12.7%. The background factors related to hemorrhagic complications were investigated but due to the insufficient number of patients, hemorrhage-associated factors could not be identified. In the previous TM alfa randomized controlled trials, no significant difference could be observed in the incidence of major bleeding compared with the placebo control [19, 20]. In patients with renal dysfunction, the TM alfa blood concentration reportedly did not increase excessively even at normal doses due to the effect of other compensatory metabolic processes such as activated elastase-mediated degradation [12, 22, 23]. The TM alfa dose is not expected to be associated with hemorrhagic complications, although further studies would be required in this field.

No previous studies have reported how TM alfa, including its dosage, could affect clinical outcomes. In this study, we showed for the first time that the normal TM alfa dose in patients with severe renal dysfunction might be a factor that improves short-term clinical outcomes without increasing adverse bleeding events. However, this study also has several limitations. First, this was a single-center, retrospective study, and the influence of potential confounders cannot be ruled out. Second, this study examined DIC resolution and 30-days-all-cause mortality but did not examine the long-term prognosis. In a retrospective study, it is difficult to determine the long-term prognosis in multiple cases, a prospective multicenter study would thus be recommended. Third, patients with an increased TM alfa dose may have been excluded if they recovered from acute kidney injury (AKI) during the DIC treatment period, which would make the reduced-dose group unfavorable for analysis as they were underdosed for TM alfa because of AKI recovery. In this study, six patients in the reduced-dose group recovered from AKI during the DIC treatment period, and all of them were alive at the 30-day follow-up. If organ damage, including kidney damage, improved early, the prognosis would be good owing to its mild severity, and the prognosis of these patients would not change with TM alfa administration [19]. In addition, none of the excluded patients had a dose increase from 130 to 380 U/kg because of AKI recovery. Therefore, the analysis of the reduced-dose group would not be disadvantageous. Fourth, the present study did not sufficiently examine bleeding complications. TM alfa safety in patients with severe renal dysfunction has not been clarified, although it would be expected that a normal dose of TM alfa would not cause an excessive concentration increase. The relationship between TM alfa dose and hemorrhagic complications in patients with severe renal dysfunction needs further investigation.

## Conclusions

In this study, we showed that a reduced dose of TM alfa in patients with severe renal dysfunction was an influential factor in DIC resolution and 30-day all-cause mortality, as were SOFA scores and pneumonia. A prospective, multicenter interventional study is warranted to validate this finding.

## Data Availability

The datasets generated and analyzed during the current study are not publicly available due to privacy concerns and institutional policy but are available from the corresponding author on reasonable request.

## Abbreviations

ICU: Intensive Care Unit
DIC: Disseminated Intravascular Coagulation
TM: Thrombomodulin
CCr: Creatinine Clearance
SOFA: Sequential Organ Failure Assessment
JAAM-DIC: Japanese Association for Acute Medicine DIC Diagnostic Criteria
ATIII: Antithrombin III
SIRS: Systemic Inflammatory Response Syndrome
FFP: Fresh Frozen Plasma
PC: Platelet Concentrate
PMX-DHP: Polymyxin B Immobilized Fiber Column Direct Hemoperfusion
OR: Odds Ratio
CI: Confidence Interval
AKI: Acute Kidney Injury
